# Reference Ranges of Cholesterol Sub-Fractions in Random Healthy Adults in Ouagadougou, Burkina Faso

**DOI:** 10.1371/journal.pone.0116420

**Published:** 2015-01-22

**Authors:** Alice T. C. R. Kiba Koumaré, Linda P. L. Sakandé, Elie Kabré, Issaka Sondé, Jacques Simporé, Jean Sakandé

**Affiliations:** 1 Health Department, Laboratory of Biochemistry, University of Ouagadougou, Ouagadougou, Burkina Faso; 2 Clinic Philadelphie of Ouagadougou, Clinical Laboratory, Ouagadougou, Burkina Faso; 3 Regional Blood Transfusion Center of Ouagadougou, Ouagadougou, Burkina Faso; 4 Centre de Recherche Biomoléculaire Pietro Annigoni (CERBA), Laboratoire de Biologie et Génétique (LABIOGENE), Centre Médical Saint Camille, Ouagadougou, Université de Ouagadougou, Ouagadougou, Burkina Faso; CEA, FRANCE

## Abstract

In Burkina Faso, the values that serve as clinical chemistry reference ranges are those provided by European manufacturers’ insert sheets based on reference of the Western population. However, studies conducted so far in some African countries reported significant differences in normal laboratory ranges compared with those of the industrialized world. The aim of this study was to determine reference values of cholesterol fractions in apparently normal adults in Burkina Faso that could be used to better assess the risks related to cardiovascular diseases. Study population was 279 healthy subjects aged from 15 to 50 years including 139 men and 140 women recruited at the Regional Center of Blood Transfusion of Ouagadougou, capital city of Burkina Faso (West Africa). Exclusion criteria based on history and clinical examination were used for defining reference individuals. The dual-step precipitation of HDL cholesterol sub-fractions using dextran sulfate was performed according to the procedure described by Hirano. The medians were calculated and reference values were determined at 2.5^th^ and 97.5^th^ percentiles. The median and upper ranges for total cholesterol, LDL cholesterol, total HDL cholesterol and HDL2 cholesterol were observed to be higher in women in comparison to men (p <0.05). These reference ranges were similar to those derived from other African countries but lower than those recorded in France and in USA. This underscores the need for such comprehensible establishment of reference values for limited resources countries. Our study provides the first cholesterol sub-fractions (HDL2 and HDL3) reference ranges for interpretation of laboratory results for cardiovascular risk management in Burkina Faso.

## Introduction

In Burkina Faso, the values that serve as clinical chemistry reference ranges are those provided by European manufacturers’ inserts sheets based on reference of the Western population. However, studies conducted so far in some African countries reported significant differences in normal laboratory ranges compared with those of other African countries and industrialized world [1–5]. Differences were even observed between urban and rural populations [3,6,7].These could be due to a number of reasons including differences in geographical locations, climate, food habits, life style, socio-economic status, races or ethnic, heredity, methodology adopted for the measurement etc. [5,7,8]. It is therefore important to evaluate whether the use of European’s lipids reference ranges to interpret Burkina Faso population’s results would not increase the dyslipidemia diagnosis’ errors and probably delay the beginning of cardiovascular risks prevention. Indeed, cardiovascular diseases have become the leading cause of death worldwide and particularly in Africa [9]. The best control strategy is based on early identification of risks factors especially those modifiable in particular dyslipidemia. This suggests that the development of reference ranges specific for Burkina Faso population is critical for interpretation of laboratory test results and provision of quality services in the health care delivery. The aim of this study was to determine reference values of cholesterol fractions in apparently normal adults in Burkina Faso that could be used to better assess the risks related to cardiovascular diseases. Indeed, the invert relation between the cardiovascular accident and the concentration of HDL, particularly its sub-fractions HDL2 is well established [10,11].

## Materials and Methods

### Subjects

The study was carried out from September 2012 to February 2013 in Ouagadougou, capital city of Burkina Faso (West Africa). Subjects were recruited at the Regional Center of Blood Transfusion of Ouagadougou and biological analyses was performed at the laboratory of Philadelphie Private Clinic of Ouagadougou. Study population was 279 healthy subjects aged between 15 to 50 years including 139 men and 140 women. To search for possible variations related to age, the study population was divided into three homogeneous subgroups (A: 15–25 years, B: 26–36 years, C: 37–50 years). This distribution reflects the changes in the height and the weight depending on the age as reported by Henny et al. [12].

The following exclusion criteria [3,4,8] based on history and clinical examination were used for defining reference individuals: diabetes melitus, dyslipedemias, cardiovascular diseases, treatment with any lipid medication (statines, nicotinic acid, fibrate, resins), renal disease, endocrine disorders, liver obstruction, excessive body weight (BMI>25), smoking, alcohol consumption, pregnancy, strenuous exercise, caffeine intake and use of oral contraceptive pill. HIV positive subjects (naïve and on HAART) were also excluded using Burkina Faso algorithm (2 Serological tests: Determine^R^ and SDBioline^R^)because of the risk of the virus and the antiretroviral treatment on lipids profile.

### Cholesterol fractions measurement

After an overnight fast, venous blood was collected on a dry tube for biochemical tests. Serum was separated by centrifugation at 3000 g for 10 min at 4°C, stored at -80°C and analyzed within a week. The determination of cholesterol and its fractions was performed using the Roche COBAS E311 chemistry analyzer (Roche Diagnostics, Mannheim Germany). Dextran sulfate (Mr 500,000; SIGMA, France) and magnesium chloride (Mg Cl2–6H20; Merck, France) were used to prepare the solutions used to split the cholesterol. References of reagent kits used and their assay principle are shown in Table 1.

**Table 1:** References of reagent kits used and their assay principle.

Analyte	Principles of methods	Kits	Roche Diagnostics References
Se TC	Cholesterol oxydase /esterase	Cholesterol Gen.2/CHOL2	00656769
Se HDL-c	Polyanion: Cholesteroloxydase / esterase	HDL-Cholesterol plus 3rd generation HDLC3	00657768
Se LDL-c	Polyanion: Cholesteroloxydase /esterase	LDL-Cholesterol plus 2nd generation/LDL_C	00766275
Se TG	GPO/Peroxydase	Triglycerides TRIGL	00767107

Se = serum; TC = total cholesterol; HDL-c = HDL-cholesterol; LDL-c = LDL-cholesterol; TG = triglycerides

The dual-step precipitation of HDL sub-fractions was performed according to the procedure described by Hirano [10] with light modifications. The slight modification of the method was verified for its accuracy and precision by performing concurrent verification procedure (precision and accuracy studies—Table 2). To isolate total HDL-c by precipitation, a combined precipitant consisting of 100 μl (0.02 mmol/L) of dextran sulfate (Mr 500000, SIGMA, France) and 25 μl (200 mmol/L) of MnCl2 (MgCl2–6H20, MERCK, France) was added to 1 ml of serum. After 15 min of standing at room temperature, the mixture was centrifuged at 3,400 *g* for 20 min at 4°C. Aliquots of the resulting supernatant (S1) were taken for the assay of the HDL-c and precipitation of the HDL2 chosterol. The HDL2-c was precipitated by a combined precipitant consisting of 100 μl (0.02 mmol/L) of dextran sulfate (Mr 500000, SIGMA, France) and 50 μl (200 mmol/L) of MnCl2 (MgCl2–6H20, MERCK, France) added to 500 μl of supernatant (S1). After 2 h at room temperature, the mixture was centrifuged at 3,400 *g* for 20 min at 4°C. Aliquots of the resulting supernatant (S2) were taken for the assay of the HDL3-c. The measured value for total HDL-c was multiplied by 1.125 and that for HDL3 cholesterol was multiplied by 2.92 to correct for dilution by the reagents. HDL3-c was measured by the direct HDL-c homogenous assay instead of the original total cholesterol assay. The sub-fraction HDL2-c was calculated by the following formula: cholesterol HDL2-c = HDL−c–HDL3-c. We calculated the LDL-Cholesterol (in mmol/L) by using the Friedewald formula: LDL-c = total cholesterol—HDL-c—Trigycerid/2.2 [13].

**Table 2:** The accuracy and precision of the measurements and day to day coefficient of variations.

Analyte	Accuracy (n = 20)			Precision	
	Reference mean	Mean obtained	Student Test Calculed t	intra-serial CV (n = 20)	inter-serial CV (n = 20)
CT	2.87	2.81	0.83(p = 0.67)	0.56	0.98
HDL-c	0.77	0.65	0.37(p = 0.15)	1.05	0.94
LDL-c	1.54	1.39	1.67(p = 0.38)	0.98	1.56
TG	1.23	1.16	0.80(p = 0.20)	1.39	0.67

TC = total cholesterol; HDL-c = HDL-cholesterol; LDL-c = LDL-cholesterol; TG = triglycerides; CV = coefficient of variations

### Quality Control

Analysis was conducted by trained and competent personnel. The Laboratory is enrolled in a National EQA program for cholesterol and HDL fractions determination with 2 surveys per year. During the period of the experiment, the laboratory performance was within the stated acceptable limits of EQA performance as required by our National EQA program. To ensure the accuracy and precision of the test results the internal control check was done every day and Standard Deviation (SD) and coefficient of variations were calculated. The Table 2 shows an overview of the quality control material used for the evaluation of the assay along with the day to day coefficient of variations. The accuracy and precision of the measurements during the study in Table 2 were within the acceptable criteria of literature [8,10,14].

### Ethics Statement

The study protocol and consent procedure were approved by the Burkina Faso National Ethics Committee for Research Ouagadougou, Burkina Faso #2012–06–52(7-June 2012). Written informed consent was obtained from all adults and from children’s parents or legal representatives prior to conducting any study procedures. After the consent was given, personal details, as well as, epidemiological data were recorded in a paper file. All the data used in this study was anonymous.

### Statistical analysis

All calculations for determining reference ranges were based on the guidelines found in the Clinical and Laboratory Standards Institute (CLSI)[15]. Data distribution was evaluated using the Kolmogorov-Smirnov test and found to be non-Gaussian. Reference ranges were calculated using non parametric methods. The medians were calculated and reference values were determined at 2.5^th^ and 97.5^th^ percentiles. Values were compared using nonparametric Mann-Whitney test. The statistical analysis was performed using the statistical software PASW, version 18 for Windows (SPSS CPSC., Chicago, USA). Probability levels of 0.05 or less were considered significant.

## Results

The table 3 presents the reference values of the cholesterol fractions of the overall study population and by age group. Only the values of total cholesterol and LDL cholesterol were significantly higher (p <0.05) in the age group of 37–50 years. The reference values of cholesterol fractions by sex are shown in Table 4. The median and upper ranges for total cholesterol, LDL cholesterol, HDL cholesterol and HDL 2 were observed to be higher in women in comparison to men (p<0.05). Reference values by sex and age group (Table 5) indicate that in men, there was no significant difference between age groups. However, among women, there was a significant increase in total cholesterol level (p <0.05), in LDL-cholesterol level (p <0.01) and a significant decrease (p <0.05) of the HDL2-cholesterol in the age group of 37–50.

**Table 3:** Reference Ranges of cholesterol fractions [median (percentiles 2.5 et 97.5)].

Analyte	Age groups in yrs(n)			Totale (n = 279)
	15–25 (n = 83)	26–36 (n = 102)	37–50 (n = 94)	
TC (mmol/L)	4.42(2.57–5.85)	4.55(2.74–5.77)	4.93(3.32–5.91) **	4.67 (2.95–5.83)
LDL-c (mmol/L)	2.22(1.00–4.09)	2.39(1.18–4.12)	2.86(1.27–4.21) **	2.55 (1.17–4.11)
HDL-c (mmol/L)	1.18(0.70–1.69)	1.16(0.67–1.67)	1.12(0.69–1.77)	1.15 (0.70–1.69)
HDL3-c (mmol/L)	0.68(0.38–1.61)	0.68(0.41–1.01)	0.68(0.39–0.92)	0.68 (0.38–0.98)
HDL2-c (mmol/L)	0.49(0.14–1.06)	0.41(0.14–1.01)	0.39(0.16–0.97)	0.43 (0.15–1.07)

TC = cholesterol total; LDL-c = LDL-cholesterol; HDL-c = HDL-cholesterol; HDL3-c = HDL3-cholesterol; HDL2-c = HDL2-cholesterol ** = significative diffence between age groups(p<0.05)

**Table 4:** Reference Ranges of cholesterol fractions according to sex [median (percentiles 2.5 et 97.5)].

Sex	Analyte (mmol/L)				
	TC	LDL-c	HDL-c	HDL3-c	HDL2-c
Males (n = 139)	4.53(2.93–5.81)	2.35(1.18–3.99)	1.08(0.65–1.77)	0.68(0.35–0.98)	0.40(0.15–1.00)
Females (n = 140)	4.88(2.93–5.86) **	2.69(1.16–4.18) **	1.18(0.79–1.68) **	0.68(0.40–0.98)	0.49(0.14–1.07) **

TC = cholesterol total; LDL-c = LDL-cholesterol; HDL-c = HDL-cholesterol; HDL3-c = HDL3-cholesterol; HDL2-c = HDL2-cholesterol ** = significative difference between males and females (p<0.05)

**Table 5:** Reference Ranges of cholesterol fractions according to age groups and sex [median (percentiles 2.5 et 97.5)].

Analyte (mmol/L)	Males (age groups in yrs)			Females (age groups in yrs)		
	15–25 (n = 40)	26–36(n = 55)	37–50(n = 44)	15–25 (n = 43)	26–36 (n = 47)	37–50 ans (n = 50)
CT	4.45 (2.55–5.92)	4.60 (2.51–5.70)	4.67 (3.59–5.89)	4.38 (2.30–5.79)	4.87 (2.75–5.96)	5.08 (3.01–6.00) **
LDL-c	2.21 (0.91–4.10)	2.36 (1.13–3.98)	2.50 (1.26–3.92)	2.19 (1.02–4.06)	2.59 (1.11–4.15)	2.96 (1.25–4.62) ***
HDL-c	1.15 (0.69–1.69)	1.11 (0.54–1.71)	1.02 (0.60–1.80)	1.24 (0.77–1.74)	1.28 (0.72–1.68)	1.10 (0.79–1.60)
HDL3-c	0.70 (0.36–0.96)	0.66 (0.33–1.10)	0.68 (0.35–0.99)	0.66 (0.38–1.42)	0.70 (0.48–1.00)	0.70 (0.38–0.93)
HDL2-c	0.43 (0.14–1.03)	0.42 (0.16–0.84)	0.39 (0.14–1.00)	0.54 (0.10–1.10)	0.54 (0.14–1.09)	0.38 (0.16–0.86) **

C = cholesterol total; LDL-c = LDL-cholesterol; HDL-c = HDL-cholesterol, HDL3-c = HDL3-cholesterol; HDL2-c = HDL2-cholesterol

** = significative difference between age groups (p<0.05)

*** = significative difference between age groups p<0.01)

## Discussion

The reference ranges of total cholesterol were slightly higher than those reported by a similar study in Ouagadougou, Burkina Faso in 2004 [3]. In a decade, the lower limit increased from 2.6 to 2.95 mmol/L and the upper limit from 5.61 to 5.83 mmol/L. This could be due to the habit change related to the increasing urbanization of African cities. Indeed, the differences in lipid levels between urban and rural areas were reported in other studies [5,6]. This finding underscores the necessity of periodic establishment of reference values to reflect changes in populations’ biological profiles.

Compared to other African countries, the total cholesterol reference ranges were similar to one derived from Ivory Coast [1], Kenya [7], Tanzania [7] and Mozambique [16]. On the other hand, total cholesterol levels in Burkina were higher than those from Benin[4], Ghana [17] and Nigeria [5].Compared to Indian values, total cholesterol values were close to those of Goswami [18] and Gupta[6] but lower than those of Madhumita [8] and Sairam [19]. Our values were lower than those recorded in France [20] and in USA [7]. This confirms that there is great variations of plasma lipid level in different populations and usually are affected by foods habits, life style, socioeconomic status, races, heredity etc.

In women, the total cholesterol was significantly higher (p <0. 05) according to authors in Benin [4], Ghana [17], Chad [21], Kenya [7], Nigeria [5], Mozambique [16] and India [8] except the French study [20] which reported no significant total cholesterol level between males and females. We also found an increase in total cholesterol level with age according to authors in Burkina Faso [3] and Cameroon [2]. The LDL values followed the same evolution with total cholesterol [8,18].

The values of total HDL were similar to those previously established in Burkina Faso [3]. However, they are lower than those established in Cameroon [2], Ghana [17], Mozambique [16], India [6,8,18] and France [20,22]. These reference values of total HDL were significantly higher in women as reported by other authors in Africa, India and Europe [8,16,18,20,22]. The influence of age and sex is probably due to hormones that affect levels of HDL-c by regulating the activity of two enzymes the endothelial lipoprotein lipase and hepatic lipase involved in the production and catabolism of HDL-c [23]. Estrogen tends to raise HDL-c and testosterone rather tends to reduce HDL-c [24]. This translates into a higher cardiovascular risk in men compared to women before menopause. After menopause, cardiovascular risk is equivalent between the two sexes and even greater in women over 75 years [25].

The reference values of HDL3-c observed in the study population were lower than those in London [26] and French values [20,22] but similar to the Brazilian values [27]. In addition, our HDL3-c values showed no significant difference according to age and sex. This agrees with Atger [22] and Roche [20] in France. On contrary in Brazil, Sodré [27] reported a significant increase in HDL 3-c in women.

The values of HDL2-c in our study are similar to Western values [20,22,26] and those of Brazil [27]. Comparison by gender showed a significant increase in HDL2-c in women (p = 0.02). This is in agreement with other authors [20,22,27]. The study showed a significant decrease of HDL2-c with age (p <0. 05). This is according to authors who reported that HDL2-c has a stronger negative correlation with age than total HDL and HDL3-c [26]. They reported also that HDL2-c were 20% higher in subjects over 55 years of age compared with those were less than 55 years of age. This confirms the increased cardiovascular risk with age.

Through this work, the reference ranges can’t be transposed from one country to another. It therefore appears that the use of higher Western lipid reference ranges for the interpretation of results in Burkina Faso may cause late detection of dyslipidemia that could delay the prevention of cardiovascular diseases. Furthermore the use of Western reference ranges can induce subnormal results requiring other additional explorations at the expense of poor people without health insurance in our country. Similarly, the use of Western reference ranges for clinical management could lead to unnecessary treatment. In Kenya a study has shown that strict adherence to reference ranges developed in industrialized countries for HIV vaccine and/or treatment trials could exclude many healthy Kenyans from participating in these studies [7].

In conclusion, the cost of non-quality probably induced on the health care system and family budgets by using inadequate reference ranges underscores the need for such comprehensible establishment of reference values for limited resources countries.

This study provides the first cholesterol sub-fractions (HDL2 and HDL3) reference ranges for interpretation of laboratory results for cardiovascular risk management in Burkina Faso.

## Supporting Information

S1 Dataset (XLSX)
